# Evaluation of the sensitivity and synergistic effect of *Trichoderma reesei* and mancozeb to inhibit under *in vitro* conditions the growth of *Fusarium oxysporum*

**DOI:** 10.1080/19420889.2020.1829267

**Published:** 2020-10-20

**Authors:** María Fernanda Gonzalez, Freddy Magdama, Luis Galarza, Daynet Sosa, Christian Romero

**Affiliations:** a Escuela Superior Politécnica del Litoral, ESPOL, Centro de Investigaciones Biotecnológicas del Ecuador (CIBE), Guayaquil, Ecuador; b Facultad de Ingeniería Química, Universidad de Guayaquil, Guayaquil, Ecuador; c Facultad de Ciencias de la Vida (FCV), Escuela Superior Politécnica del Litoral, ESPOL, Guayaquil, Ecuador

**Keywords:** Biological control, fungicide, *Fusarium oxysporum*, *Trichoderma reesei*, minimum inhibitory concentration, synergistic effect

## Abstract

Trichoderma

is a saprophytic, soil-borne fungus with a worldwide distribution that has been extensively studied due to their capacity to synthesize secondary metabolites with antimicrobial activity, parasitize other fungi and directly interact with plant roots, inducing resistance to disease and tolerance to abiotic stresses. *Fusarium* wilt caused by the soil-inhabiting fungus *Fusarium oxysporum* is considered one of the most important diseases that affect banana cultivars. Currently, more environmentally friendly alternatives to control this disease are being proposed, these strategies include the application of low doses of synthetic fungicides and the use of biocontrol agents such as *Trichoderma* or *Xylaria*. Thus, this study aimed to evaluate under *in vitro* conditions the synergistic effect of the biological control agent *T. reesei* C2A combined with low doses of mancozeb to inhibit the mycelial growth of *F. oxysporum* F1. To perform the synergistic essays, 0.1 mg/mL of mancozeb was suspended in PDA plates, then plugs of *T. ressei* C2A were placed at the center of the Petri dishes, the plates were incubated for 7 days at 28°C. Results showed that the mycoparasitic capacity of the biocontrol strain to inhibit the mycelial growth of *F. oxysporum* F1 was enhanced approximately 36% compared to the control plates. Although these results are promising, future studies under greenhouse and field conditions are necessary to corroborate the effectiveness of this approach.

## 1. Introduction

Banana is a tropical fruit that grows in more than 130 countries, it ranks amongst the world’s most valuable primary agricultural commodity due to its nutritional properties [1,2]. In the sixties, banana industry experienced dramatic losses due to *Fusarium* wilt caused by the soil-borne fungus *Fusarium oxysporum* [3,4]. *Fusarium* wilt was detected in Ecuador in 1936 at the United Fruit plantations in Tenguel [5]. *F. oxysporum* penetrates the plant through the tertiary roots, then passes into the rhizome vascular system and pseudostem and invades the xylem vessels. The fungus produces conidia, which is carried along the vascular bundles where they start new areas of infection, causing their obstruction preventing water transport and reducing nutrients uptake [6]. It also secretes toxins that induce wilting by altering cell metabolism [7], withering by *F. oxysporum* remains a latent concern as it represents a threat to the production of banana in Ecuador, causing considerable manufacturing and exporting losses.

The main agricultural method to control this pathogen is based on the application of synthetic fungicides or sterilants, including mancozeb, methyl bromide and quaternary ammonium, which can be applied as powdered, emulsions, granulated, or solutions [8]. Mancozeb is a dithiocarbamate, non-systemic agricultural fungicide with high spectrum of biological activities against a wide range of pathogenic fungi including ascomycetes, oomycetes and basidiomycetes [9,10]. Although the application of synthetic fungicides to control the growth of phytopathogens has been satisfactory in most cases, evidence suggests that synthetic compounds including benomyl, chlorothalonil, captan, mancozeb, maneb and propiconazole can cause adverse health effects on humans and the environment [11,12]. Thus, more environmentally friendly alternatives to control the growth of phytopathogens are currently being proposed. These strategies include the application of low doses of synthetic fungicides combined with the inoculation of biological agents such as nonpathogenic fungi (*Trichoderma, Clonostachys* and *Xylaria*) or bacteria (*Bacillus, Lysinibacillus* and *Solibacillus*) [13,14]. It has been documented that these microorganisms exhibit strong antagonistic interactions (i.e. antibiosis, parasitism and competition for space and nutrients) with plant pathogens to maintain they growth at a lower density that would occur in the absence of biological competitors [14,15].

*Trichoderma* is considered as a potential ecological alternative to transform agricultural systems highly dependent on synthetic inputs into sustainable and more productive systems [16]. Under *in vitro* and greenhouse conditions, several strains of *Trichoderma* were able to inhibit the growth of a wide range of phytopathogens [17–20]. *Trichoderma* species are also considered important plant growth-promoting fungi (PGPF) as they significantly facilitate plant growth and development by increasing solubilization of organic compounds increasing nutrient efficacy, releasing plant-growth stimulatory agents and inducing systemic resistance [21–25]. This study aimed to establish under *in vitro* conditions an environmentally friendly alternative to control the mycelial growth of the phytopathogen causing banana *Fusarium* wilt. This strategy consisted of evaluating in solid media the synergistic interaction of the biological control agent *T. reesei* C2A combined with low doses of mancozeb to inhibit the mycelial growth of *F. oxysporum* F1.

## 2. Materials and methods

### 2.1. Fungal strains and fungicide used

This study was conducted at the *Centro de Investigaciones Biotecnológicas del Ecuador* (CIBE) from the *Escuela Superior Politécnica del Litoral* (ESPOL). The fungal strains *T. reesei* C2A (biological control) and *F. oxysporum* F1, F2 and F3 (phytopathogens) were obtained from CIBE’s Microbial Culture Collection. Before carrying out the sensitivity and synergistic essays, the strains were reactivated three times in Potato Dextrose Agar (PDA) and grown for 7 days at 28°C, or until the mycelium sporulated. The selection of the pathogenic strain used in this study was conducted quantitatively using a Kruskal–Wallis test with multiple comparisons (p < 0.05) finding that F1 diameter growth was statistically different that F2 and F3 while confronted to *T. reesei* C2A for 2 days (Results not shown). Mancozeb was purchased from a certified commercial house and was used to perform the sensitivity and synergistic assays.

### 2.2. Macroscopic and microscopic characterization of the pathogenic and beneficial fungi

The identity of the fungal strains was confirmed by macroscopic and microscopic characteristics such as the presence of conidia, mycelium coloration and presence of hyphae. Three Petri dishes containing PDA were inoculated with 8 mm plugs of both fungi, the plates were incubated for 7 days at 28°C before performing the macroscopic and microscopic identification.

### 2.3. In vitro experimental design

#### 2.3.1. Dual culture plate assay

The mycoparasitic capacity of *T. reesei* C2A to control the growth of *F. oxysporum* F1 was evaluated by the dual culture plate essay as described elsewhere [26,27]. The tests consisted of the inoculation of 8 mm plugs containing agar and mycelium of *T. reesei* C2A on one side of a Petri dish containing PDA, on the other side of the plate, a mycelial plug of *F. oxysporum* F1 was inoculated maintaining a distance of 6 cm between each disc. Plates were then incubated for 7 days at 28°C. *T. reesei* C2A mycoparasitism was measured daily using the antagonistic capacity index described in Table 1.

**Table 1.** Scale used to measure the antagonistic capacity of *T reesei* C2A

Antagonistic degreea	Antagonistic capacity (Pathogen-Antagonist)
0	No invasion of the surface of the colony of *F. oxysporum*
1	Invasion of 1/4 of the surface of the colony of *F. oxysporum*
2	Invasion of 1/2 of the surface of the colony of *F. oxysporum*
3	Total invasion of the surface of the colony of *F. oxysporum*
4	Total invasion of the surface of the colony of *F. oxysporum* sporulation on it

a Adapted from [28]

#### 2.3.2. Minimum inhibitory concentration assays

The application of different doses of mancozeb against the beneficial strain *T. reesei* C2A and the pathogenic strain *F. oxysporum* F1 was evaluated by establishing the minimum inhibitory concentration. Mancozeb was suspended in sterile-distilled water and added to Petri dishes containing molten PDA to achieve final concentrations of 0.1, 0.01, 0.001 and 0.0001 mg/mL, respectively. Once the agar solidified, 8 mm plugs containing agar and mycelium of each fungus were transferred to the center of the plates. The plates were incubated for 7 days at 28°C, mycelial growth of the colonies were measured in two directions (vertically and horizontally) and reported as the average value of the two measurements [19].The experiment was performed in triplicate and carried out three times to ensure the reproducibility of the results.

#### 2.3.3. Determination of the percentage inhibition of diameter growth (PIDG)

The percentage inhibition of diameter growth (PIDG) was used to determine the ability of mancozeb to inhibit the mycelial growth of both the pathogen *F. oxysporum* F1 and the beneficial fungus *T. reesei* C2A [29]. The PIDG was estimated by measuring the diameter growth of the fungal strains inoculated in Petri dishes containing 15 mL of PDA supplemented with 4 different concentrations of mancozeb (0.1, 0.01, 0.001 and 0.0001 mg/mL) comparing to the growth of the positive controls. Cultures were incubated for 7 days at 28°C and mycelia measuring growth was performed every 24 hours.

$$PIDG\left(\%\right)=\frac{Diameter\;of\;the\;sample-diameter\;of\;the\;positive\;control}{diameter\;of\;the\;positive\;control}\times 100$$

#### 2.3.4. Synergistic assays using mancozeb

Synergistic inhibition of the mycelial growth of the pathogen *F. oxysporum* F1, was assessed based on the protocol described by [30]. *In vitro* tests were performed in PDA Petri dishes supplemented with 0.1 mg/mL of mancozeb. 15 mL of molten PDA were transferred to a sterile 15 mL Falcon tube, an aliquot of mancozeb from a stock solution (3 mg/mL) was added to the tube to obtain a final concentration of 0.1 mg/mL (v/v), the agar was then poured into the Petri dish and gently shaken to get a homogenous mixture. Once the agar solidified, 8 mm plugs containing agar and mycelia of 5-day fungi cultures of *T. reesei* C2A and *F. oxysporum* F1 were inoculated at each side of the Petri dish at 1.5 cm from the edge and maintaining 6 cm of distance between each disc. Plates were then incubated for 7 days at 28°C. The percentage of inhibition of *F. oxysporum* F1 was calculated using the equation described by [30,31]. All experiments were performed in triplicate and independently replicated three times.

$$\%\;I=\frac{A1-A2}{A1}*100$$

where %I = inhibition percentage (%), A1 = area of the Petri dish in mm^2^ covered by *Fusarium oxysporum* F1, control plate that was not added the commercial fungicide (mancozeb), A2 = area of the Petri dish in mm^2^ covered by *Fusarium oxysporum* F1 co-inoculated with *T. reesei* C2A and 0.1 mg/mL of mancozeb.

### 2.4. Data analysis

Macroscopic and microscopic identification was performed by examining the morphological characteristics of the mycelial growth. Qualitative data obtained from the dual culture plate essays was analyzed using the observational ranking reported by [32]. Quantitative measurements from the *in vitro* essays were recorded in internal records in the laboratory and then imported to Excel. Measurements of the growth of the mycelial diameter were reported in cm and included the 8 mm agar disc. Initial data entry of the mycelial growth from the macroscopic essays showed that 12 values were missing due to random contamination of the Petri dishes. The missing values were imputed using the means substitution method [33].

Measurements of the mycelial growth from the dual culture plate essays were compared to the growth of the positive controls for the first 3 days because as of day 4, the mycelia of both fungi were overlapped. The mycelial growth reduction was reported as the percentage of growth inhibition and compared to the positive control. The percentage of inhibition of *F. oxysporum* F1 was calculated by comparing the mycelial growth of the positive controls to the growth of *F. oxysporum* F1 when is co-cultured in the same Petri dish with the biocontrol strain *T. reesei*.

he minimum inhibitory concentration of mancozeb was determined by measuring the mycelial growth of each fungal strain separately for 7 days, comparison of the mycelial growth in cm was performed using an ANOVA test in R Studio [34]. This analysis was conducted independently for the four concentrations of mancozeb (0.1, 0.01, 0.001, 0.0001 mg/mL). All mean values were reported with standard deviation in centimeters (cm), and mean plots were used to illustrate the mycelial growth over time. The variables tested were the mean diameter growth for 7 days using the Tukey method with 95% confidence. Different letters were used to show a significant difference over the days, and the more distant letters were used to select the concentration that was chosen to carry out the synergy test.

To study the evolution over time of the two co-cultured fungal strains in Petri dishes supplemented with 0.1 mg/mL de mancozeb, the PIDG of *T. reesei* against *F. oxysporum* was calculated by an experimental factorial design with repetitions using multiple comparisons with the Tukey method. All statistical tests were done with a significance of 5%, using R Studio, P-values lower than 0.05 were considered to show significant differences. Finally, growth over time was tested by comparing the diameter growth from day one to day three, and the time in hours from day 1 to day 3, using the equation described by [31]:

$$TC=\frac{C2-C1}{T2-T1}$$

Where: TC = growth rate (cm/h); C1 = initial growth (cm); C2 = final growth (cm); T1 = initial time (h); T2 = final time (h)

## 3. Results

### 3.1. Macroscopic and microscopic characterization of the pathogenic and beneficial fungal strains

**Figure 1.**
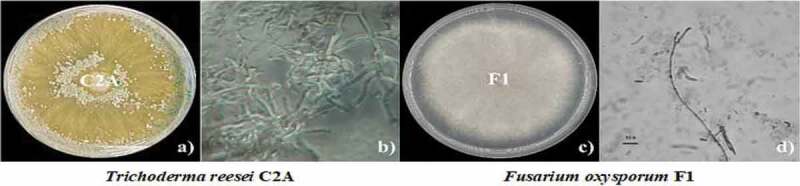
Macroscopic and microscopic characteristics of *T. reesei* C2A (a and b) and *F. oxysporum* F1 (c and d) in PDA, plates were incubated at 28°C for 7 days

Results showed that both fungi had well-defined macroscopic and microscopic characteristics. *T. reesei* exhibited a rapid growth in PDA and after about 60 to 72 hours of being incubated at 28°C, the mycelia covered the entire diameter of the 9 cm Petri dish (Figure 1). Typical growth characteristics of *T. reesei* C2A included white cottony hyphae without aerial mycelium, yellowish-greenish conidia and change of the medium coloration due to the production of a yellowish pigment. Micromorphology characteristics such as hyaline conidiophores, long primary branch and a short secondary branch were observed. *T. reesei* C2A exhibited whitish mycelia which grew approximately at a rate of 3.41 cm a day. After 2 days of incubation, mycelia color changed from whitish to yellowish and covered the entire diameter of the Petri dish as of day 3, mycelia color turned greenish-grayish with a smooth appearance exhibiting greenish ellipsoidal conidia. Macroscopic characteristics of *F. oxysporum* F1 started with an initial growth rate of 1.50 cm at day 1, with a steady increase up to 8.69 cm on day 7, it did not produce diffusible pigments, its vegetative and aerial mycelia were whitish and pinkish, respectively, macroconidia and microconidia were analyzed to identify the species of the pathogenic strain.

### 3.2. Dual culture plate assay

**Figure 2.**
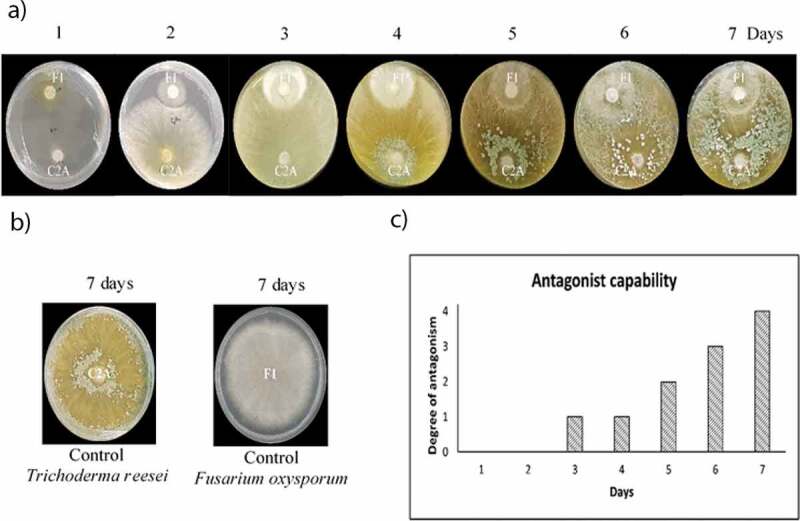
Assessment of antagonistic activity of *T.* *reesei* C2A against *F.* *oxysporum* F1. a) Dual plate culture essay in PDA exhibiting the antagonistic capacity of *T.* *reesei* C2A to inhibit the growth of *F.* *oxysporum* mycelia. b) Growth of positive controls in PDA. C) Antagonist capability of *T.* *reesei* C2A using a 0–4 scale as stated by [28]

The antagonistic effect of *T. reesei* C2A against *F. oxysporum* F1 was observed after 3 of incubation as fungal colonies started competing for space and nutrients and the mycelia of the biocontrol strain grew and sporulated over the pathogen, *T. reesei* C2A fully covered the Petri dish including ¼ of *F. oxysporum* F1 (Figure 2). *Trichoderma* mycelia changed color from greenish to grayish and invaded 50 to 100% of the *Fusarium* colony. *T. reesei* C2A experienced an exponential mycelial growth from 3.41 cm at day 1 to 9 cm at day 2. In contrast, *F. oxysporum* F1 showed a steady mycelial growth (1.50-day 1, 2.92-day 2, 4.24-day 3, 5.37-day 4, 6.90-day 5, 7.83-day 6 and 8.69-day 7). Growth inhibition was calculated using radial growth measurements of days 1, 2 and 3, radial growth of days 4 to 7 could not be measured as *Trichoderma* mycelia overgrown in the plates. Inhibition increased over time, from 59% on day 1 to 62% on days 2 and 3 (Table 2). These results suggest that *T. reesei* C2A exhibited strong antagonistic activity against *F. oxysporum* F1, inhibiting mycelial growth and preventing sporulation. Thus, *T. reesei* C2A could be used as a potential biological control to prevent *Fusarium* wilt in banana plantations.

**Table 2.** Diameter growth (30 observations) of *T.* *reesei* C2A and *F.* *oxysporum* F1 in Petri dishes incubated at 28°C, data were daily recorded for 3 days

Day	Mean growth (SD) in cm of *Trichoderma reesei* C2A	Mycelial growth (SD) in cm of the positive control	Mean growth (SD) in cm of *Fusarium oxysporum* F1	Mycelial growth (SD) in cm of the positive control	Percentage of antagonistic effect between C2A and F1	p-value^a^
1	3.48 (0.38)	3.41 (0.58)	1.43 (0.1)	1.50 (0.25)	59%	0.08501
2	7.13 (3.31)	9 (0)	2.73 (0.1)	2.92 (0.21)	61%	0.09154
3	9 (0)	9 (0)	3.41 (0.2)	4.24 (0.26)	62%	0.1695

^a^difference in mycelial growth of *Fusarium oxusporum* F1, it was evaluated using a non-parametric hypothesis test

### 3.3. Minimum inhibitory concentration assays

**Figure 3.**
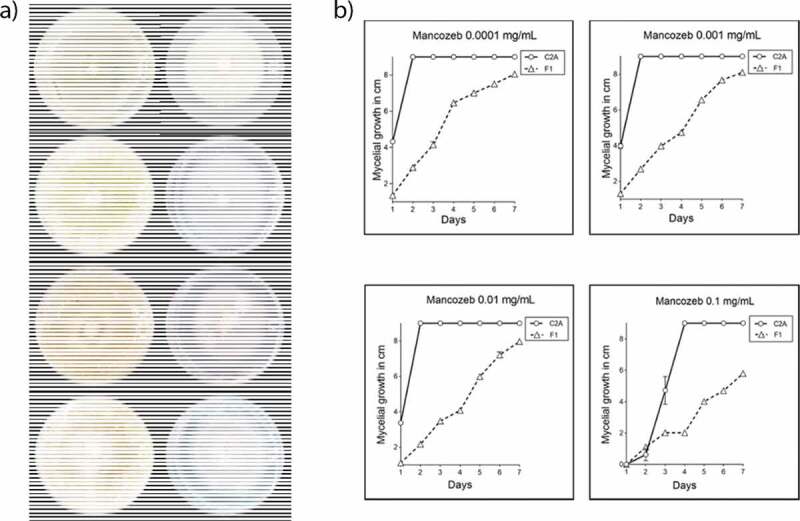
a) Mycelial growth of *T. reesei* C2A and *F. oxysporum* F1 in 9 cm Petri dishes supplemented with 4 different concentrations of mancozeb (0.1, 0.01, 0.001 and 0.0001 mg/mL) after 7 days of incubation at 28°C. b) Plot of means of the mycelial growth of *T. reesei* C2A and *F. oxysporum* F1 after 7 days of incubation at 28°C

Mycelial growth was reported as the average of the vertical and horizontal measures in centimeters. Using the Tukey’s method, we determined that during the 7-day assay there were significant differences between the mycelial growth of the biocontrol agent and the phytopathogenic strain. The mycelial growth of *T. reseei* C2A was not significantly affected by any of the concentrations used (0.1, 0.01, 0.001 and 0.0001 mg/mL) (P < 0.0001). Although when mancozeb concentrations of (0.1, 0.01, 0.001 and 0.0001 mg/mL) were added to the culture medium, the mycelial growth of *F. oxysporum* F1 was reduced by 33.2, 8.3, 6.8 and 7.4%, respectively, compared to the positive control (Table 3). The growth of the phytopathogen was significantly reduced (33.2%, P < 0.0001) only when 0.1 mg/mL of the fungicide were suspended in the medium. As the growth of *F. oxysporum* F1 was significantly affected at 0.1 mg/mL, this concentration was selected to perform the synergistic assays (Figure 3).

**Table 3.** Growth diameter in cm (SD) of *T.* *reesei* C2A and *F.* *oxysporum* F1 in experiments with four different concentrations of mancozeb

Incubation time	Growth in cm				
***T. reesei* C2A**	**Control**	**0.1 mg/mL**	**0.01 mg/mL**	**0.001 mg/mL**	**0.0001 mg/mL**
Day 1	3.41 (0.58)	0.00 (0) ^a^	3.38 (0.14) ^a^	3.98 (0.31) ^a^	4.33 (0.25) ^a^
Day 2	9.00 (0)	0.63 (0.86) ^a^	9.00 (0) ^b^	9.00 (0) ^b^	9.00 (0) ^b^
Day 3	9.00 (0)	4.72 (1.98) ^b^	9.00 (0) ^b^	9.00 (0) ^b^	9.00 (0) ^b^
Day 4	9.00 (0)	9.00 (0) ^c^	9.00 (0) ^b^	9.00 (0) ^b^	9.00 (0) ^b^
Day 5	9.00 (0)	9.00 (0) ^c^	9.00 (0) ^b^	9.00 (0) ^b^	9.00 (0) ^b^
Day 6	9.00 (0)	9.00 (0) ^c^	9.00 (0) ^b^	9.00 (0) ^b^	9.00 (0) ^b^
Day 7	9.00 (0)	9.00 (0) ^c^	9.00 (0) ^b^	9.00 (0) ^b^	9.00 (0) ^b^
***F. oxysporum* F1**	**Control**	**0.1 mg/mL**	**0.01 mg/mL**	**0.001 mg/mL**	**0.0001 mg/mL**
Day 1	1.50 (0,25)	0.00 (0) ^a^	1.11 (0.23) ^a^	1.31 (0.05) ^a^	1.36 (0.12) ^a^
Day 2	2.92 (0,21)	1.11 (0.02) ^b^	2.17 (0.23) ^b^	2.68 (0.07) ^b^	2.90 (0.12) ^b^
Day 3	4.24 (0.26)	2.02 (0.1) ^c^	3.48 (0.10) ^c^	3.97 (0.04) ^c^	4.15 (0.38) ^c^
Day 4	5.37 (0.43)	2.02 (0.1) ^c^	4.08 (0.07) ^d^	4.73 (0.20) ^d^	6.46 (0.28) ^d^
Day 5	6.90 (0.78)	4.01 (0.1) ^d^	5.99 (0.30) ^e^	6.56 (0.21) ^e^	7.00 (0.27) ^e^
Day 6	7.83 (0.63)	4.70 (0.1) ^e^	7.21 (0.39) ^f^	7.67 (0.32) ^f^	7.51 (0.05) ^f^
Day 7	8.69 (0.59)	5.80 (0.2) ^f^	7.97 (0.04) ^g^	8.10 (0.23) ^g^	8.05 (0.25) ^g^

### 3.4. Percentage inhibition of diameter growth (PIDG)

**Figure 4.**
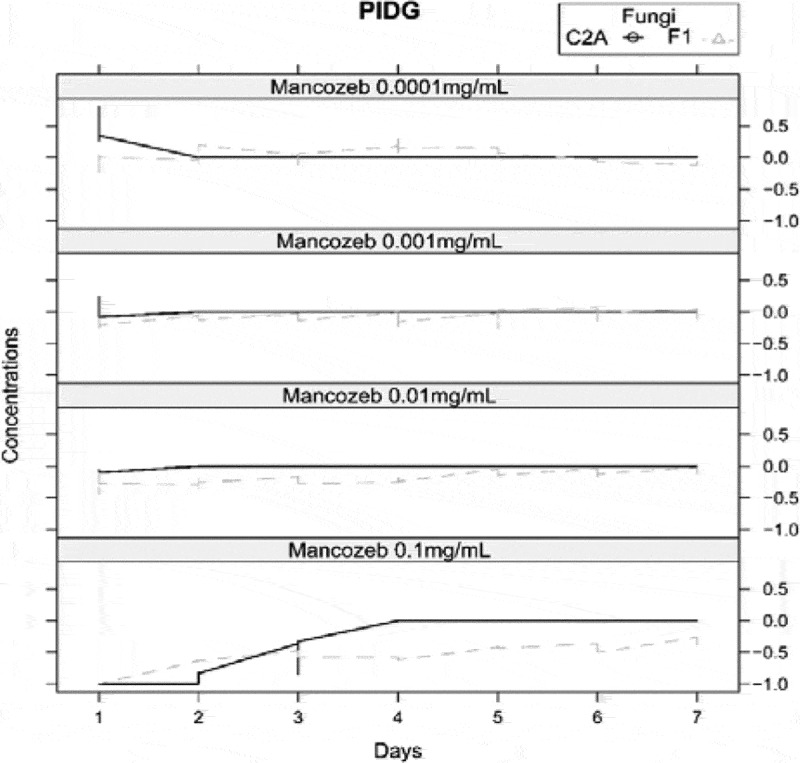
Graph of percentage inhibition of diameter growth of *T.* *reesei* C2A and F. oxysporum F1 in PDA with 4 different concentrations of mancozeb

The interaction between both fungi was measured by PIDG in PDA with 4 different concentrations of mancozeb. Figure 4, shows that over time the biocontrol agent grew faster than the pathogen strain in presence of 0.1 mg/mL of mancozeb (Figure 4). The Figure indicates that only at 0.1 mg/mL of mancozeb, the percentage of inhibition of *Fusarium* had negative values from day 1 till day 7, meaning that the mycelial growth of *F. oxysporum* F1 is significantly inhibited compared to the positive control, whereas since day 4, the growth of *T. reesei* C2A was not affected by the presence of the fungicide.

### 3.5. Synergistic tests between T. reesei C2A and 0.1 mg/mL of mancozeb against F. oxysporum F1

**Figure 5.**
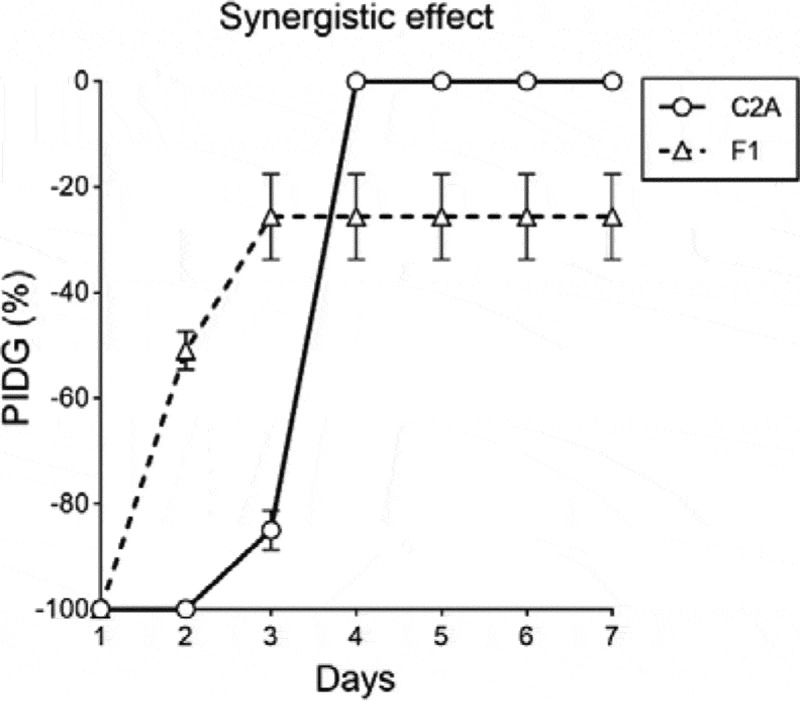
Synergistic effect of *T.* *reesei* C2A against *F.* *oxysporum* F1 in PDA plates supplemented with 0.1 mg/mL of mancozeb

**Figure 6.**
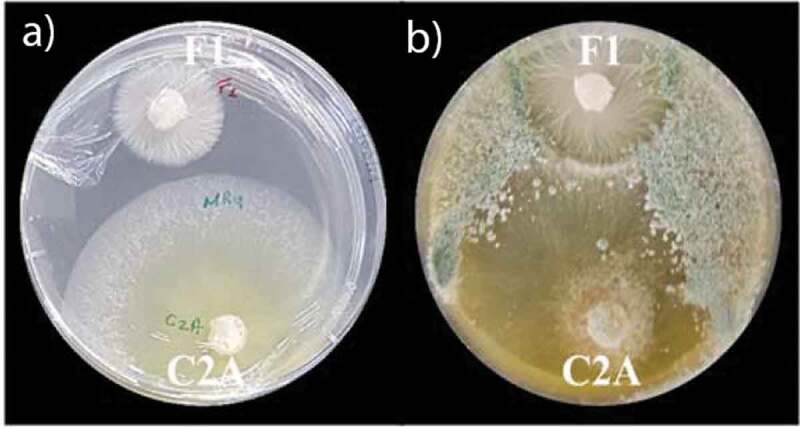
Synergistic effect between the biocontrol strain *T.* *reesei* C2A and 0,1 mg/mL of mancozeb to inhibit the growth of *F.* *oxysporum* F1. a) Picture was taken after 3 days of incubation at 28°C. b) Picture was taken after 7 days of incubation at 28°C

The synergistic tests of *T. reesei* C2A against *F. oxysporum* F1 in PDA plates supplemented with 0.1 mg/mL of mancozeb, showed that the first day of the essay, the mycelial growth of the phytopathogen was completely inhibited (100%). As of day 2, growth inhibition of *F. oxysporum* F1 decreased to 51% and *T. reesei* C2A mycelia started overgrowing toward the pathogenic strain (Figure 5). By day 3, *F. oxysporum* F1 mycelial growth inhibition was 36% compared to the positive control and growth inhibition of *T. reesei* C2A was reduced only 10% compared to the control. The following days, the biocontrol strain continued overgrowing toward *F. oxysporum* F1 till day 7, when the Petri dish was totally covered by *Trichoderma* (Figure 6).

These results suggested that *T. reesei* C2A could be used in combination with low doses of the fungicide as an environmentally friendly alternative to inhibit under *in vitro* conditions the growth of banana phytopathogens.

## 4. Discussion

*Trichoderma* species are soilborne organisms associated with the roots of plants that have been widely used in agriculture for their potential to control plant diseases. Species of *Trichoderma* are recognized for their mycoparasitic and antibiosis capability to inhibit the mycelial growth of various pathogens including *F. oxysporum, F. solani, Alternaria alternata, Botrytis cinerea* and *Rhizoctonia solani* [35,36]. In this study, the biocontrol strain *T. reesei* C2A and the pathogenic strain *F. oxysporum* F1 were characterized based on their macroscopic and microscopic features as reported elsewhere [37–40]. *T. reesei* C2A showed a typical whitish cottony mycelium that changed to a photosensitive greenish-grayish color after sporulation (i.e. after 3 or 4 days of incubation). The production of diffusible pigments, which are characteristic of *T. reesei* isolates, were detected at the bottom of the plates. Furthermore, distinguishing conidiophores, conidia and intercalary phialides were observed too [41]. *F. oxysporum* F1 on the other hand, exhibited concentric rings, hyaline conidiophores with pyramidal ramifications and fixed solitary accessories as the ones reported by [38,42]. Finally, ITS (Internal transcribed spacer) molecular markers were used to completed the taxonomic identity of the fungal strains (data not shown). The dual culture plate assays and synergy tests allowed us to monitor for 7 days under *in vitro* conditions the antagonistic and synergistic capacity of *T. reesei* C2A to inhibit the mycelial growth of *F. oxysporum* F1. The evolution over time of the percentage of inhibition in the confrontational essays shown during the first 3 days, confirmed the ability of *T. reesei* C2A to inhibit the growth of *F. oxysporum* F1. On the first day, the PIDG of *F. oxysporum* F1 was 59% compared to the positive control, inhibition then increased to 61% and 62% on the second and third day, respectively, demonstrating the mycoparasitic mechanism of the biocontrol strain overgrowing toward the phytopathogen. Similar results were reported by [43–45], who using four different strains (T29, T1, T2 and T3) of *T. reesei* demonstrated that they were able to inhibit in a high percentage the mycelial growth of *F. oxysporum* f. sp. *cubense, F. oxysporum* f. sp. *cicero* and *F. oxysporum* f. sp. *melongenae. T. reesei* isolates are known to activate a large battery of secondary metabolites and/or enzymes including plant cell wall degrading enzymes (CWDEs) which degrade the cell wall of soil-plant pathogens [46,47].

Although the current methods to control *Fusarium* wilt are based on the application of synthetic fungicides such as mancozeb, methyl bromide and quaternary ammonium, the pathogen is not always eliminated and might continue sporulating in necrotic tissues due to its saprophytic capacity [8,48,49]. The use of these fungicides is not an economically and environmentally sustainable practice because their overuse have led to serious environmental pollution problems, generating pathogen resistance and leaving toxic residues on fruits [50]. Thus, more environmentally friendly alternatives to control the development of phytopathogenic fungi are currently being proposed to reduce the potentially harmful effects of the continuous application of synthetic pesticides and fertilizers on agricultural lands [51,52]. These strategies involve the synergistic application of low doses of synthetic fungicides combined with biocontrol agents such as the nonpathogenic fungi *Trichoderma, Clonostachys* and *Xylaria* [13,14]. Recent studies under *in vitro* conditions have shown that when mancozeb is added to the culture medium in concentrations lower than 5 mg/mL, the mycelial growth of *Trichoderma* is not significantly inhibited [53,54]. Furthermore [55], tested the tolerance of 26 *Trichoderma* isolates against 4 pesticides and evaluated their antagonistic capacity against the sheath blight pathogen of rice *Rhizoctonia solani*. The authors reported that the pesticide pyrethroid significantly enhanced the growth of all *Trichoderma* isolates and the strains *T. reesei* and *T. longibrachiatum* were the most effective in inhibiting the growth and the sclerotial formation of *R. solani*. Furthermore [30], were able to quantify under *in vitro* conditions the synergistic interaction of the synthetic fungicide Captan 50® and *Trichoderma asperelleum* T8a, their results showed that by using this integrated alternative, the mycelial growth of *Colletotrichum gloeosporioides*, the causal agent of anthracnose in mango, was inhibited 99% compared to the positive control. The authors suggested that this approach could help reduce economic and environmental problems in the field as lower amounts of Captan 50® might be used to control the growth of *C. gloeosporioides*. The synergistic essays performed in this study using 0.1 mg/mL of mancozeb combined with the fungal strain *T. ressei* C2A, demonstrated that the mycoparasitic capacity of the biocontrol strain was enhanced when lower concentrations of the fungicide were applied to the culture medium. As depicted in Figure 6, *T. ressei* C2A grew faster than *F. oxysporum* F1 as of the second day of incubation and continued growing faster till the third day. As of day 3, *T. ressei* C2A overgrowth toward the pathogen inhibiting 36% of the mycelial growth of *F. oxysporum* F1 compared to the control plates. Our results strongly suggest that the biocontrol agent *T. ressei* C2A could be used in combination with the synthetic fungicide mancozeb to promote a synergistic effect that inhibits in a higher percentage of the growth of the phytopathogens. Although these results are promising, future studies under greenhouse and field conditions are necessary to corroborate the effectiveness of this approach.

## 5. Conclusion

The biocontrol strain *Trichoderma reesei* C2A obtained from the Microbial Culture Collection held at the Centro de Investigaciones Biotecnológicas del Ecuador, exhibited under *in vitro* conditions mycoparasitic activity, reducing 62% the mycelial growth of *F. oxysporum* F1after 3 days of incubation at 28°C. The synergistic essays demonstrated that when the growth medium is suspended with low concentrations of mancozeb (0.1 mg/mL) combined with plugs of the biocontrol strain *T. ressei* C2A, the mycoparasitic capacity of the biocontrol strain was enhanced approximately by 36% compared to the positive control. These results strongly suggest that *T. ressei* C2A could be used in combination with mancozeb as an environmentally friendly alternative to control the growth of *F. oxysporum*, the causal agent of *Fusarium* wilt.
